# Hippocampal Oligodendrocytes Regulate Mossy Fiber Development Involved in Epileptic Responses

**DOI:** 10.1007/s12264-025-01452-x

**Published:** 2025-07-17

**Authors:** Chunxia Jiang, Yunan Hu, Feng Zhang, Mengsheng Qiu, Xiaofeng Zhao

**Affiliations:** 1 Institute of Developmental and Regenerative Biology, Zhejiang Key Laboratory of Organ Development and Regeneration, College of Life and Environmental Sciences, Hangzhou Normal University, Hangzhou, 310036 China; 2 Quzhou Affiliated Hospital of Wenzhou Medical University, Quzhou People’s Hospital, Quzhou, 324000 China

**Keywords:** Oligodendrocyte, Differentiation, Myelin-associated glycoprotein, Tropomyosin-related kinase B, Epilepsy

## Abstract

Although oligodendrocytes (OLs) are known to form the myelin sheath around neuronal axons for the saltatory conduction of action potentials, recent studies have suggested that OLs also modulate neuronal function and plasticity. In the present study, we found that OL maturation deficiency in *Myrf*-CKO mice caused spontaneous epileptogenesis and resulted in death. To further investigate the association between OL development and epilepsy, we examined the *Adamts4* KO mouse line, which has a mild OL differentiation phenotype in the hippocampus. As a result, the differentiation defect in the mutant hippocampus reduced the expression of myelin-associated glycoprotein and lessened its inhibition of the dephosphorylation of phosphorylated tropomyosin-related kinase B, which is associated with retarded adolescent hippocampal mossy fiber development and higher susceptibility to epileptogenesis in adulthood. More importantly, enhancing differentiation by orally administered clemastine rescues the defective mossy fiber development in the early postnatal period and attenuates epilepsy susceptibility in adults. Together, these results strongly suggest that an OL differentiation defect in the hippocampus may contribute to susceptibility to epilepsy in adults.

**Supplementary Information:**

The online version contains supplementary material available at 10.1007/s12264-025-01452-x.

## Introduction

Temporal lobe epilepsy (TLE) is the most common adult form of epilepsy, in which a hippocampal onset accounts for at least 80% [1, 2]. Despite advances in the pharmacological treatment of epilepsy, in more than 30% of patients, the seizures still can’t be completely controlled [3, 4]. One prominent theory of epileptogenesis is the "excitatory-inhibitory" imbalance in hippocampal neurons. Neural circuits in the hippocampus balance excitatory and inhibitory activity and disruptions of this balance cause neurological disorders such as epilepsy [5]. The neural circuit in the hippocampus consists of four components: dentate gyrus (DG), cornu ammonis 1 (CA1), CA3, and entorhinal cortex. The DG sends projections to the pyramidal cells in CA3 through mossy fibers [6], the axon bundles originating from granule cells [7]. Since the supra-pyramidal mossy fiber (SMF) projection is much more stable than the infra-pyramidal mossy fiber (IMF) system, newly-formed mossy fibers preferentially join the IMF system. The relative distance of the IMF to SMF is used to assess MF development during hippocampal neural circuit formation [8]. Since MF development plays a significant role in seizure generation and hippocampal excitability, an altered MF length ratio implies a changed threshold of epileptogenesis in the mouse hippocampus [9].

Oligodendrocytes (OLs) form myelin sheaths wrapping around neuronal axons to ensure the rapid and efficient conduction of nerve impulses, which plays an essential role in neural circuit function [10]. Equally importantly, OLs also provide trophic and metabolic support for ensheathed axons and neurons [11]. In addition, OLs expressing proteins such as myelin-associated protein (MAG) and NOGO are inhibitory for the growth and regeneration of axons [12] and modulate neuronal function and plasticity. In the hippocampus, the differentiated OL-specific proteins MAG and myelin basic protein (MBP) start to be detectable around P10 and rapidly increase in the second postnatal week [13, 14]. Normal development of OLs and their interaction with neurons are crucial to well-structured and functioning neural circuits. Aberrant OL development impairs brain functioning and is frequently associated with neuropsychiatric diseases, including epilepsy, schizophrenia, major depression, and some cognitive disorders [15].

In the present study, we report that *Myrf*-CKO mice lacking mature OLs developed recurrent spontaneous behavioral seizures resulting in death. To further investigate the association between OL differentiation and epilepsy, we examined the *Adamts4* KO mouse line, which with a mild phenotype of OL differentiation. It has been demonstrated in our recent research that ADAMTS4 enhances OL differentiation by cleaving NG2 proteoglycan and attenuating PDGFRα signaling [16]. Interestingly, we found that *Adamts4* mutant mice with impaired hippocampal OL differentiation in the juvenile stage become more susceptible to drug-induced epilepsy in adulthood, even though myelin is restored. Mechanistically, a defect in OL differentiation in the mutant hippocampus reduced the expression of MAG protein and lessened its inhibition of pTRKB dephosphorylation, which is associated with retarded hippocampal mossy fiber development and higher susceptibility to epileptogenesis. Furthermore, enhancement of OL differentiation in *Adamts4* mutants with the drug clemastine significantly improved hippocampal mossy fiber development and increased the threshold of epileptogenesis in adults, demonstrating the potential of enhancing OL differentiation at early postnatal stages as a novel strategy to abate or prevent epileptogenesis in adulthood.

## Materials and Methods

### Animals

Lines for *Adamts4*-KO, *Plp*-eGFP, *Olig1*^Cre^, and *Myrf*-CKO mice have been described [16–20]. *Adamts4* ^−/−^; *Plp*-eGFP mutantswere produced by sequential breeding of *Adamts4* knockout mice with *Plp*-eGFP mice. PCR-genotyping was carried out using genomic DNA with the following primers: *Adamts4* knockout-p1 (5’-GGG TGG GAT TAG ATA AAT GCC TGC TCT-3’), *Adamts4* knockout-p2 (5’-GGA CAC GGG ATG GAC CCT CTA GAT G-3’), *Adamts4* knockout-p3 (5’-ACA TGG AGG ACT CAG TGT GGC CCA C-3’); *Plp*-eGFP-p1(5’-ACG TAA ACG GCC ACA AGT TC-3’), *Plp*-eGFP-p2(5’-GGG GTG TTC TGC TGG TAG TG-3’); *Olig1*^Cre^-p1(5’-CGT TAG TGA AGG GCG CCC CGG GTC G-3’), *Olig1*^Cre^-p2(5’-CGC TAG AGC CTG TTT TGC ACG TTC AC-3’); *Myrf*-CKO-p1(5’- GGG AGG GGG CTT CAA GGA GTG T-3’), and *Myrf*-CKO-p2(5’-CCC CCA GCA TGC CGA TGT ACA C-3’). *Adamts4*-null allele resulted in a 495-bp band, while the control allele produced a 326-bp band; the *Plp*-eGFP allele resulted in a 500-bp band; the *Myrf* -CKO allele resulted in a 667-bp band, while the control allele produced a 460-bp band; the *Olig1*^Cre^ allele resulted in a 550-bp band. In this study, all animal protocols followed ethical guidelines and were approved by the Laboratory Animal Center, Hangzhou Normal University, and the Animal Ethics Committee of Hangzhou Normal University, China (permit number: 2024043; approved on 29 February 2024).

### Immunofluorescence Staining

Mice were anesthetized and fixed with 4% paraformaldehyde (PFA). Brain tissues were isolated and transferred into 25% sucrose for equilibration on the next day. Tissues were embedded in OCT, and frozen sections were cut at 16 μm on a cryostat. For immunofluorescence, sections were rinsed with phosphate buffer saline (PBS) three times for 5 min each and blocked in PBS containing 5% goat serum and 0.1% Triton X-100 for 1 h at 37 ℃. Sections were then incubated overnight at 4 ℃ with the following primary antibodies: anti-Calbindin (Cell signaling Technology, 13176, 1:100), anti-MAG (Millipore, MAB1567,1:500), anti-NOGO (Abconal, A1752,1:500), anti-MBP (Abcam, ab7349, 1:500), anti-ASPA (Oasis Biofarm, OB-PRT005, OB-RB037, 1:500), anti-MYRF (Oasis Biofarm, OB-PRB007, 1:500), or anti-SOX10 (Oasis Biofarm, OB-PGP001, 1:2000). On the next day, secondary antibodies were incubated at room temperature for 1 h. The secondary antibodies were: Goat anti-Rabbit IgG (H+L) (Invitrogen, A11012, A11034,1:3000), Goat anti-Mouse IgG1 (Invitrogen, A21125, A21121, 1:3000), Goat anti-Rat IgG (H+L) (Invitrogen, A11007, A11006, 1:3000), and Goat anti Mouse IgG (Sigma, 76085, 1:2000). Tissues were then washed three times in PBS for 5 min each and mounted with Mowiol mounting medium for fluorescence microscope observations.

### Timm Staining

Mice were deeply anesthetized and perfused intracardially with PBS, followed by 0.4% sodium sulfide in 0.9% saline and 4% PFA. The brain was harvested and cut at 16 μm on a cryostat. The sections were stained in the dark for 45 min at 27 ℃ in a solution containing 30% gum Arabic, 0.2 mol/L citrate buffer, 0.15 mol/L hydroquinone, and 0.085% silver nitrate. After washing with dd H_2_O for 1 min, the slides were mounted for bright-field microscope observations.

### Western Blotting

The hippocampus was isolated from mice and lysed in RIPA lysis buffer (Beyotime, P0013B) with Protease Inhibitor Cocktail (Sigma, P8340). Protein concentration was measured by a bicinchoninic acid protein assay kit (Thermo, 23225). The total protein was subjected to electrophoresis in a 10% SDS-PAGE gel and then transferred to a PVDF membrane. The membrane was blocked with 5% non-fat milk at room temperature for 1 h and then incubated with primary antibody at 4 ℃ overnight. The sources and dilutions of primary antibodies were as follows: anti-β-actin (ABclonal, AC026, 1:10000), anti-Phospho-GluR1 (S845) (HUABIO, ET1701-28, 1:700), anti- GluR1-N terminus, clone RH95 (Millipore, MAB2263, 1:3000), anti-PSD95 (Oasis, OB-PGP053, 1:1000), anti-vGLUT1 (Bioss, bs-11167R, 1:1000), anti-TRKB (Abconal, A19832, 1:1000), anti-pTRKB (CST, 4168, 1:1000). β-actin served as the internal control. On the next day, the membrane was washed 3 times for 10 min each in TBST and incubated with a secondary antibody conjugated to HRP for ECL detection.

### Gold Myelin Staining Kit

Frozen sections were cut at 16 μm and rehydrated in ddH_2_O for 2 min, then transferred to pre-warmed Gold myelin staining solution (Oasis Biofarm, BK-AC001) and incubated in a 45 ℃ hybridization oven until the finest myelinated fibers emerged. The sections were rinsed in ddH_2_O for 2 min. Sodium thiosulfate solution was added and incubated at 45 ℃ for 3 min. The sections were then rinsed 3 times in ddH_2_O for 2 min each and cover-slipped with Mowiol mounting medium for bright-field microscope observations.

### Quantification of Glutamate in the Hippocampus

The hippocampus was removed from Control or *Myrf*-CKO mice at P15 and immediately frozen at −80 ℃. A glutamate assay kit (Sigma, MAK004) was used to measure glutamate concentration. Briefly, tissue was homogenized in 100 μL assay buffer and centrifuged at 13,000 × g for 10 min to remove insoluble material. The supernatant was filtered through a 10-kDa molecular weight cut-off spin filter (Biovision). For measurement, 90 μL of sample, 8  μL of glutamate developer, and 2 μL of glutamate enzyme mix were added into a 96-well plate (Jet Biofil, TCP011096), and then incubated for 30 min at 37 ℃. The absorbance was measured at 450 nm with a microplate reader (Bio-Rad). Concentrations of glutamate were calculated according to the reference standard curve.

### Surgical Implantation of EEG Electrodes and Recording

Bipolar electrodes were stereotaxically implanted into the CA3 area of the hippocampus (AP: −2.37 mm; ML: −1.70 mm; V: −2.92 mm) in 8-week-old mice under anesthesia. A skull screw implanted overlying the somatomotor area of the cortex served as a ground electrode. After 7 days of recovery from surgery, an electroencephalogram (EEG) was continuously recorded for 30 min in freely-moving mice as a baseline. The mice received an intraperitoneal injection of kainic acid (KA; Abcam, ab120100, 25 mg/kg). Then, EEG was recorded for an additional 2 h after KA treatment. EEG was recorded using head-mounted preamplifiers with 24× amplification and 0.37 Hz high-pass filtering (Bio-Signal Technologies). The electrical signal was digitized by Medusa from Biosignal, and sampled at 1000 Hz with a 50 Hz digital notch filter using Athena software.

### Pharmacological Seizure Induction and Behavioral Analysis

Eight-week-old male mice received intraperitoneal injections of KA (Abcam, ab120100, 25 mg/kg). Mice's behaviors were recorded every 5 min and continuously monitored for more than 120 min. The grade scale for seizure activity was according to a modified Racine scale as described previously [21].

### Electrophysiology

*Olig1*^Cre^; *Myrf* CKO mice were sacrificed by cervical dislocation at P15. Coronal slices (300 μm) were cut and removed to a chamber superfused with oxygenated artificial cerebrospinal fluid (ACSF, pH 7.4). Miniature excitatory postsynaptic currents (mEPSCs) were recorded in pyramidal neurons of the CA3 region in the hippocampus at least 10 min after 1 μmol/L TTX and 100 μmol/L picrotoxin were added to the normal ACSF. For miniature inhibitory postsynaptic current (mIPSC) recording, 1 μmol/L TTX and 10 μmol/L DNQX (glutamatergic synaptic transmission) were added into the ACSF. Data were analyzed using the Mini Analysis Program (Version 6.0.3, Synaptosoft, USA) and Clampfit (Version 10.3.1.5, Molecular Devices, LLC).

### Drug Treatments

Clemastine (MCE, HY-B0298) was dissolved in ddH_2_O at 1 mg/mL. Control or *Adamts4*-null pups were either treated daily with vehicle ddH_2_O or clemastine (10 mg/kg) by oral gavage from postnatal Day 7 (P7) to P15 and analyzed at P15, P30, and P60. Brain tissues were fixed and sectioned for histochemical analysis at P15 and P30. Pharmacological seizure induction and behavioral analyses were performed at P60.

### Statistical Analysis

Two-tailed Student’s *t*-test was used for comparisons of the two groups. Differences among multiple groups were compared using one-way ANOVA followed by Dunnett's multiple comparisons test. *P* < 0.05 was considered to be statistically significant (*n* ≥ 3). Error bars indicate SME.

## Results

### *Myrf*-CKO Mice Die of Spontaneous Epilepsy Around Postnatal Day 15

**Fig. 1:**
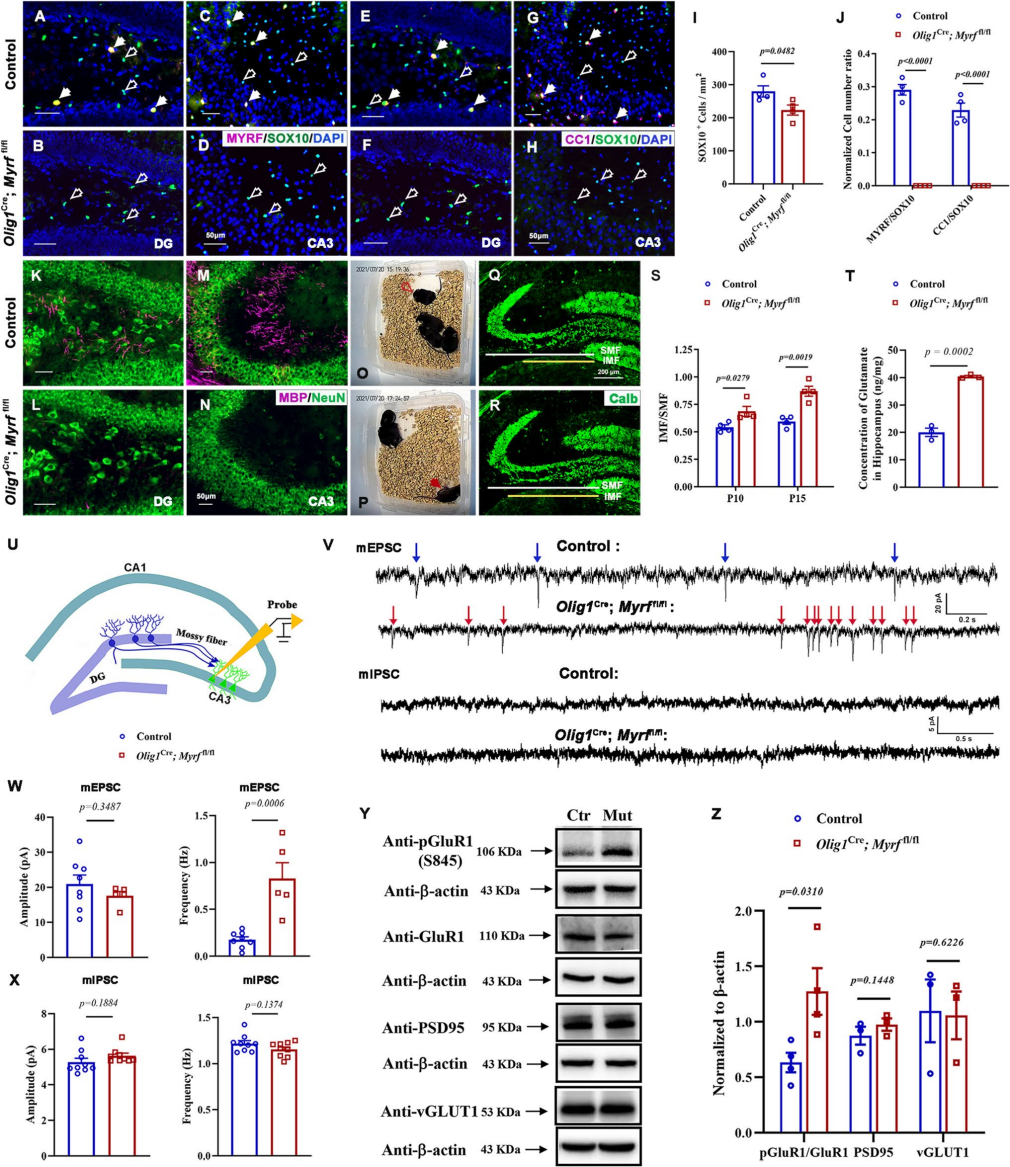
*Myrf* deletion influences oligodendrocyte differentiation in the hippocampus and causes spontaneous epilepsy in mice. **A–D** Representative images showing the co-immunostaining of the pan-oligodendrocyte marker SOX10 (green) with differentiating oligodendrocyte MYRF (red) in the DG (**A**, **B**) and CA3 (**C**, **D**) of P15 hippocampus. SOX10^+^ MYRF^+^ oligodendrocytes are indicated by arrows, and SOX10^+^ MYRF^-^ cells by empty arrows. Scale bars, 50 μm. **E**–**H** Representative images showing the co-immunostaining of SOX10 (green) with the differentiated oligodendrocyte marker CC1 (red) in P15 hippocampus. SOX10^+^ CC1^+^ oligodendrocytes are indicated by arrows, and SOX10^+^ CC1^-^ cells by empty arrows. Scale bars, 50 μm. **I** The numbers of SOX10^+^ oligodendrocytes per mm^2^ in hippocampal sections. *n* ≥ 3, *P =* 0.0482. **J** The numbers of MYRF^+^ or CC1^+^ cells among SOX10^+^ oligodendrocytes in the hippocampus. *n* ≥ 3. *P* values are annotated on the bar graphs. **K**–**N** Co-immunofluorescence raised against anti-MBP and anti-NeuN to detect mature oligodendrocytes and neurons in the DG (**K**,** L**) and CA3 (**M**, **N**) region of *Olig1* ^Cre^; *Myrf*-CKO mouse hippocampus at P15. Scale bars: 50 μm. **O**, **P** Representative screenshots from continuous, real-time recorded mouse behavior.** O** Normal behaviors. **P** *Myrf*-deficient mice appear to show tonic seizure behaviors (red arrowhead). Littermate control mice are distinguished by ear tags. **Q**, **R** Immunofluorescence raised against anti-Calbindin in the hippocampus of *Olig1* ^Cre^; *Myrf*-CKO mice at P15. IMF, infra-pyramidal mossy fiber tract; SMF, supra-pyramidal mossy fiber tract. Scale bars: 200 μm. **S** Normalized ratio of IMF to SMF at P10 and P15 in *Olig1* ^Cre^; *Myrf*-CKO mouse hippocampus. *n* ≥ 3. *P* values are annotated on the bar graphs. **T** Glutamate concentration in the hippocampus by the enzymatic assay from control or *Myrf*-CKO mice at P15. *n* = 3, *P* = 0.0002. **U–X** *Myrf* deletion impairs synaptic transmission. **U** Schematic of the whole-cell patch clamp recording site in *ex vivo* hippocampal slices. **V** Sample recordings (3 s) of miniature excitatory postsynaptic currents (mEPSCs) and 5 s of miniature inhibitory postsynaptic currents (mIPSCs) recorded from CA3 pyramidal neurons of control and mutant hippocampus at P15. **W**, **X** The average amplitude and frequency of mEPSCs (**W**) (*n* = 8 cells from 4 control mice, 5 cells from 3 mutant mice, *P* < 0.05) and mIPSCs (**X**) (*n* = 9 cells from 3 control mice, 9 cells from 3 mutant mice, *P *< 0.05). **Y**, **Z** Western immunoblots for detection of Glutamate receptor 1 (GluR1) serine phosphorylation and total protein expression levels of GluR1, PSD95, and vGLUT1 (**Y**) quantified in **Z**. *n* = 3, *P* values are annotated on the bar graphs.

*Myrf* (myelin regulatory factor) is a transcription factor that is specifically expressed by differentiating and mature OLs and plays an essential role in CNS myelination and myelin maintenance [22]. Consistent with previous reports, in *Olig1*^Cre^; *Myrf*-CKO mice, OL differentiation was stalled and the number of mature OLs decreased; furthermore, myelin was not formed throughout the entire CNS, including the hippocampus (Figs. 1A–N and S1). Interestingly, we recorded recurrent spontaneous behavioral seizures in *Myrf*-CKO mutant mice at around P15, including shivering, myoclonic whole-body clonus, and even tonic seizures (Fig. 1O, P, see Supplementary Video). During the spontaneous seizure periods, the frequency of seizures in *Myrf*-CKO mice was about 12 per day. The recurrent seizures caused the mortality of animals, and all *Myrf*-CKO mice died by P16.

### Aberrant Mossy Fiber Development and Miniature Excitatory Postsynaptic Currents in *Myrf*-CKO Hippocampus

The aberrant mossy fiber in the dentate gyrus of the hippocampus is known to be a striking morphological alteration accompanying limbic epilepsy in both human and mouse brains [23–25]. To investigate whether the recurrent spontaneous seizures in *Myrf*-CKO mice are associated with aberrant mossy fiber development, we examined the mossy fibers in *Olig1*^Cre^; *Myrf-*CKO hippocampus by anti-Calbindin immunostaining at P10 and P15, before the first seizure onset and the critical period for neural circuit formation in the hippocampus, respectively (Fig. 1Q, R). The relative distance from the IMF to the SMF is a commonly used measurement for assessing MF development during hippocampal microcircuit formation [8]. We found that the ratio of IMF to SMF increased significantly in the mutant mice compared to the control (Fig. 1S), suggestive of an abnormal MF length ratio in *Myrf* mutants. Together, these results suggested that abnormal differentiation of hippocampal OLs affects MF development, which in turn alters the epileptic behavior in mutant mice.

It was previously shown that the first 2 postnatal weeks in rodents are a critical period in development, with multiple changes that affect the balance of excitation and inhibition in the brain [26]. To investigate whether deficiency of mature OLs may influence this balance in the hippocampus, we applied the glutamate enzymatic assay and found a significant elevation of glutamate (Fig. 1T), the primary excitatory neurotransmitter, in *Myrf*-CKO mice at P15. Next, we examined the excitatory synaptic properties of CA3 pyramidal neurons in the hippocampus at P15 by recording mEPSCs. In *Myrf*-CKO mice, we also discovered a significant increase in mEPSC frequencies, but no difference in mEPSC amplitudes, indicating enhanced excitatory synaptic activity. In addition, we found that the inhibitory properties were unchanged in mIPSCs (Fig. 1U, W). Consistent with the altered mEPSC frequencies in the hippocampus, the level of glutamate receptor 1 (GluR1) phosphorylation was augmented, while the total level of GluR1 was unchanged (Fig. 1Y, Z). Notably, the expression of postsynaptic density marker PSD-95 and vesicular glutamate transporter vGLUT1 was unchanged, suggesting the lack of a systemic insult on the excitatory synapse (Fig. 1Y, Z). However, *Myrf*-CKO didn’t influence the expression of pGluR1 in the cortex (Fig. S2). Together, these results indicated that abnormal hippocampal OL development and mature OL deficiency affect postsynaptic, phosphorylation-dependent glutamate receptor trafficking and the associated glutamatergic neurotransmission, thus increasing excitatory activity in the mutant hippocampus.

### Abnormal OL Differentiation in *Adamts4* Mutants Affects the Hippocampal Mossy Fiber Length Ratio and Reduces the Threshold of Animal Susceptibility to Epilepsy

**Fig. 2:**
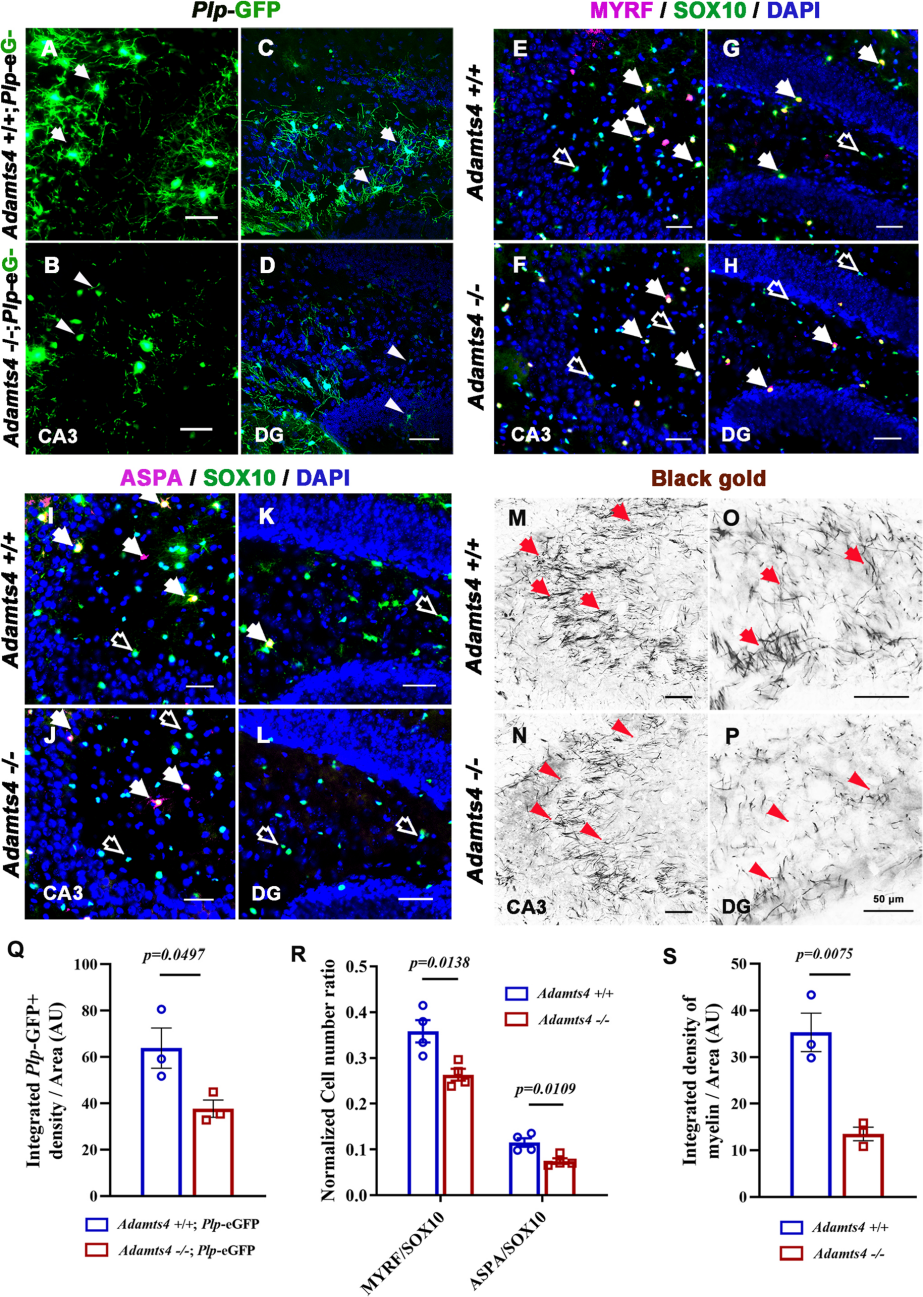
*Adamts4* deletion causes a discrepancy in oligodendrocyte differentiation in the hippocampus. **A–D** *Plp* tagged by GFP signals is lower in mutant mouse hippocampus than in the wild type at P15. Higher magnification images are shown for the CA3 (**A**, **B**) and DG (**C**,** D**) regions. Arrows, oligodendrocytes with several processes; arrowheads, oligodendrocytes with poor processes. Scale bars: 50 μm. **E–H** Representative images showing the co-immunostaining of the pan-oligodendrocyte marker SOX10 (green) with differentiating oligodendrocyte MYRF (magenta) in CA3 (**E**,** F**) and DG (**G**,** H**) of P15 hippocampus. SOX10^+^ MYRF^+^ oligodendrocytes are indicated by arrows, and SOX10^+^ MYRF^-^ cells by empty arrows. Scale bars, 50 μm. **I–L** Immunofluorescence raised against anti-ASPA and anti-SOX10 to detect mature oligodendrocytes influenced by *Adamts4* knockout in the hippocampus at P15. Scale bars: 50 μm. SOX10^+^ASPA^+^ oligodendrocytes are indicated by arrows and SOX10^+^ASPA^-^ cells by empty arrows. Scale bars, 50 μm. **M–P** Gold myelin staining of CA3 and DG of the hippocampus in *Adamts4*-KO mice at P30. Scale bars: 50 μm. **Q** Statistical analysis of the density of *Plp*-GFP^+^ cells per unit area (μm^2^) at P15. AU, Arbitrary Units. *n* = 3. *P* values are annotated on the bar graphs. **R** Number of MYRF^+^ or ASPA^+^ cells in SOX10^+^ oligodendrocytes in the hippocampus. *n* ≥ 3. *P* values are annotated on the bar graphs. **S** Density of myelin fibers per unit area (μm^2^), *n* = 3. *P* values are annotated on the bar graphs.

To further test the idea that defective differentiation of OLs in the hippocampus contributes to epilepsy, we investigated another mutant mouse line with a relatively mild phenotype in OL development. Recently, we reported that the *Adamts4* mutation causes a delay in OL differentiation in the white matter and cerebral cortex [16]. Consistent with this, there was a marked reduction of PLP-tagged GFP^+^ myelin fibers in CA3 and DG regions of the hippocampus in *Adamts4*-null mice (Fig. 2A–D, Q). Double immunostaining showed a significant decrease of newly-differentiated MYRF^+^/SOX10^+^ OLs and mature ASPA^+^/SOX10^+^ OLs in the hippocampus (Fig. 2E–H, I–L, R). In addition, gold myelin staining of P30 brain tissue revealed a remarkable decline of myelin fibers in the mutant hippocampus, particularly in the dentate gyrus and CA3 regions (Fig. 2M–P, S). Together, these results demonstrated the retarded OL maturation in the hippocampus of *Adamts4* mutants.

**Fig. 3:**
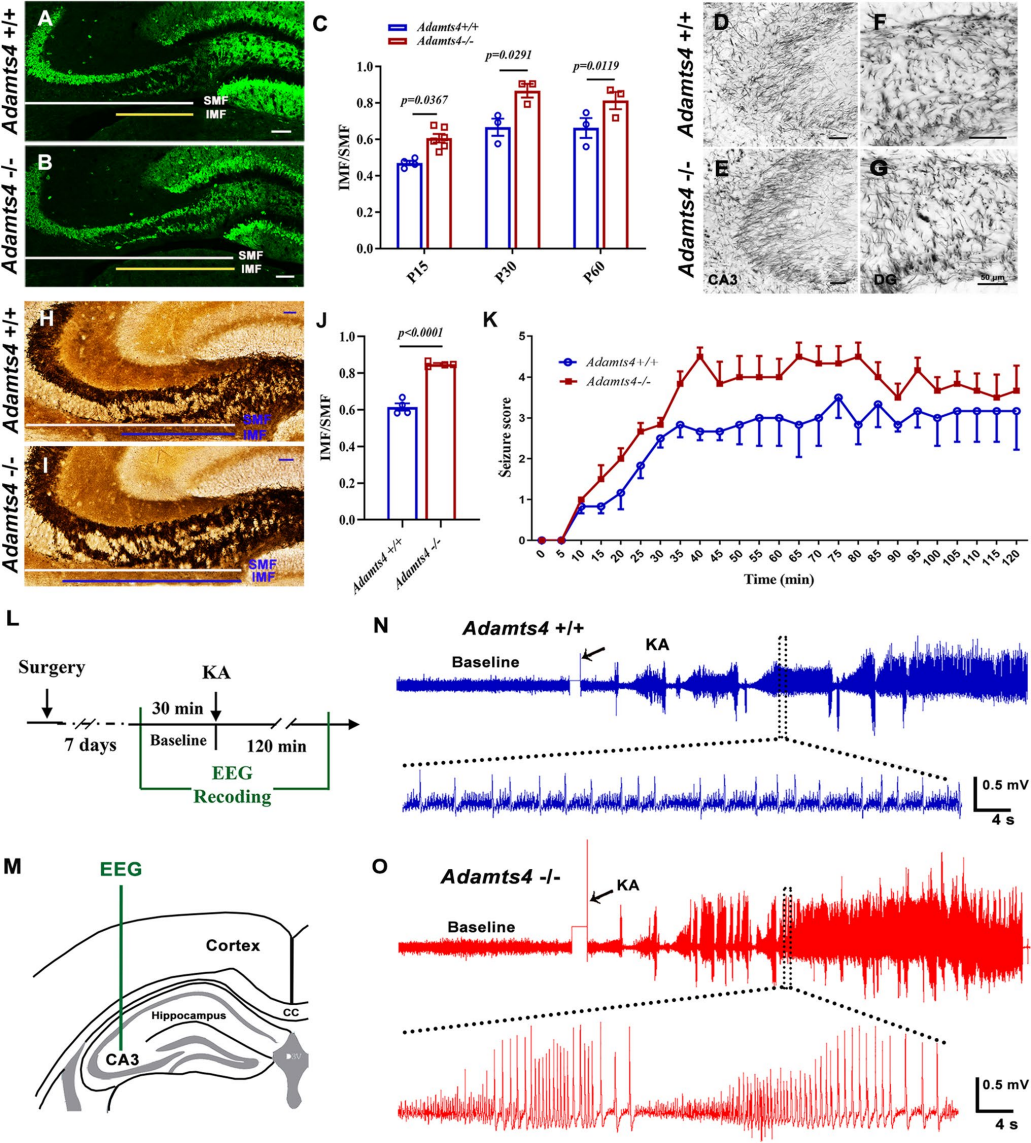
Ablation of *Adamts4* affects the mossy fiber length ratio and epileptic behavioral response in mice. **A**,** B** Immunofluorescent staining of anti-Calbindin in *Adamts4*-knockout mouse hippocampus at P15. Scale bars: 100 μm. **C** Normalized ratio of IMF to SMF at P15 to P60 in *Adamts4*-knockout mouse hippocampus (*n* = 3, *P* values are annotated on the bar graphs). IMF, infra-pyramidal mossy fiber tract; SMF, super-pyramidal mossy fiber tract. **D–G** Representative images of gold myelin staining in CA3 (**D**, **E**) and DG (**F**, **G**) of *Adamts4* mouse hippocampus at P60. Scale bars: 50 μm. **H**, **I** Timm staining of sections through the CA3 region of the *Adamts4-*KO mouse hippocampus at P15. Scale bar: 100 μm. **J** Normalized ratio of IMF to SMF of Timm staining at P15 in the *Adamts4*-knockout mouse hippocampus (*n* = 3, *P* < 0.0001). **K** Seizure scores of *Adamts4*^+/+^ and *Adamts4*^−/−^ mice within 5-min segments recorded for 120 min after KA induction (*n = Adamts4*^+/+^: 8; *Adamts4*^−/−^: 6).** L**, **M** Experiment scheme for EEG recording in the CA3 region of the hippocampus and the KA-induced seizure model. **N**, **O** Representative EEGs recorded from the hippocampus. Arrows indicate the time of KA injection.

We next investigated the possible effects of abnormal OL development on the development of hippocampal MFs and epilepsy in *Adamts4* mutants. Brain tissues were immunostained with anti-calbindin, which labels MF development from dentate granular cells in the DG molecular layer to pyramidal neurons in CA3 (Fig. 3A, B). We discovered a significantly increased length ratio of infrapyramidal mossy fibers (IMFs) to supra-pyramidal mossy fibers (SMFs) in *Adamts4* ^−/−^ mutants compared to controls at P15, P30, and P60 (Fig. 3C). In addition, Timm staining in P15 *Adamts4*-KO brain sections further confirmed increased MF length ratio in the mutant mice (Fig. 3H–J).

Although the abnormal MF length ratio had been implicated in neuronal hyper-excitation and seizure generation in the hippocampus [9, 27, 28], we did not detect apparent spontaneous seizures in *Adamts4* mutant mice as observed in *Myrf*-CKO mice. Thus, we next investigated whether the *Adamts4 -*deficient mutant could alter the animal response to induced epilepsy susceptibility in adults. In this experiment, P60 wild-type and mutant mice were intraperitoneally injected with KA, a structural analog of glutamic acid, to induce epilepsy, followed by EEG recording and behavioral scores on a modified Racine scale. Typical EEGs during seizures are shown in Fig. 3L, O. We found that *Adamts4*^−/−^ mice developed earlier seizure behavior and scored higher than wild-type littermates, indicating a reduced threshold and more severe epileptic behavior in the adult mutants (Fig. 3K).

### Decreased Expression of MAG and Increased Phosphorylation of TRKB Are Associated with Abnormal Mossy Fiber Development

**Fig. 4:**
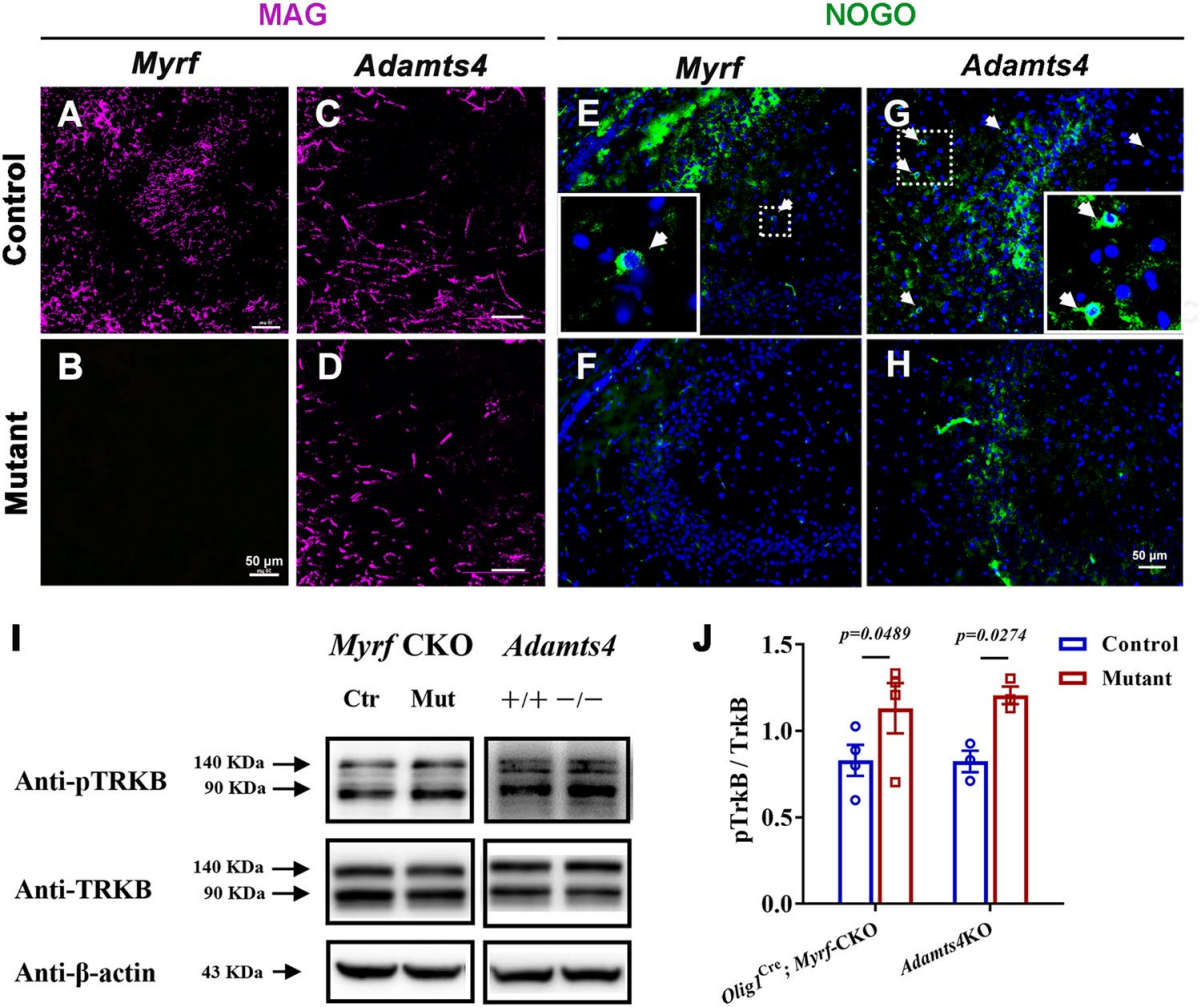
Abnormal development of oligodendrocytes decreases MAG expression, which alters the dephosphorylation of pTrkB in the *Adamts4* mutant mouse hippocampus. **A–D** Immunofluorescence raised against anti-MAG in the CA3 region of *Olig1* ^Cre^; *Myrf*-CKO (**A**, **B**) and *Adamts4*-KO (**C**, **D**) mouse hippocampus at P15. Scale bars: 50 μm. **E–H** Immunofluorescence raised against anti-NOGO in *Olig1* ^Cre^; *Myrf*-CKO (**E**, **F**) and *Adamts4*-KO (**G**, **H**) mice at P15. Scale bars: 50 μm. Higher magnification images are shown in the boxes. **I**, **J** Immunoblots and statistical analysis of the pTRKB and TRKB protein expression in the hippocampus of mice with abnormally developing oligodendrocytes at P15.

How could impaired OL development promote MF outgrowth? Previous studies demonstrated that Nogo and MAG modulate axon growth and regeneration [29–32]. Reduced expression of myelin-associated glycoprotein (MAG), potentially resulting from impaired oligodendrocyte differentiation, may disrupt the appropriate regulation of axon outgrowth.Therefore, we next compared the levels of these proteins in the mutants with naïve OLs. As expected, the protein levels of MAG and Nogo were significantly diminished in *Olig1*^Cre^; *Myrf*-CKO, and *Adamts4*-KO mutant mice (Fig. 4A–H).

Previous studies demonstrated that paired immunoglobulin-like receptor B (PIRB) is a high-affinity receptor for MAG [33]. For instance, PIRB and MAG form a complex with the trophic factor receptor tyrosine kinase B (TRKB) and subsequently recruit the SHP1 and SHP2 phosphatases. The resulting TRKB dephosphorylation suppresses neurite outgrowth [34]. Immunoblotting analyses revealed that the level of pTRKB was significantly elevated in P15 mutant hippocampal tissue, whereas the level of total TRKB protein remained unchanged (Fig. 4I, J). This result is consistent with our hypothesis that OL differentiation deficiency in the mutant hippocampus reduces MAG expression and enhances pTRKB phosphorylation, leading to increased neurite outgrowth of MFs and consequently a higher susceptibility to epileptogenesis.

### Drug Mediated Enhancement of Hippocampal Oligodendrocyte Differentiation Reduces Epileptic Responses in Adult Mice

**Fig. 5:**
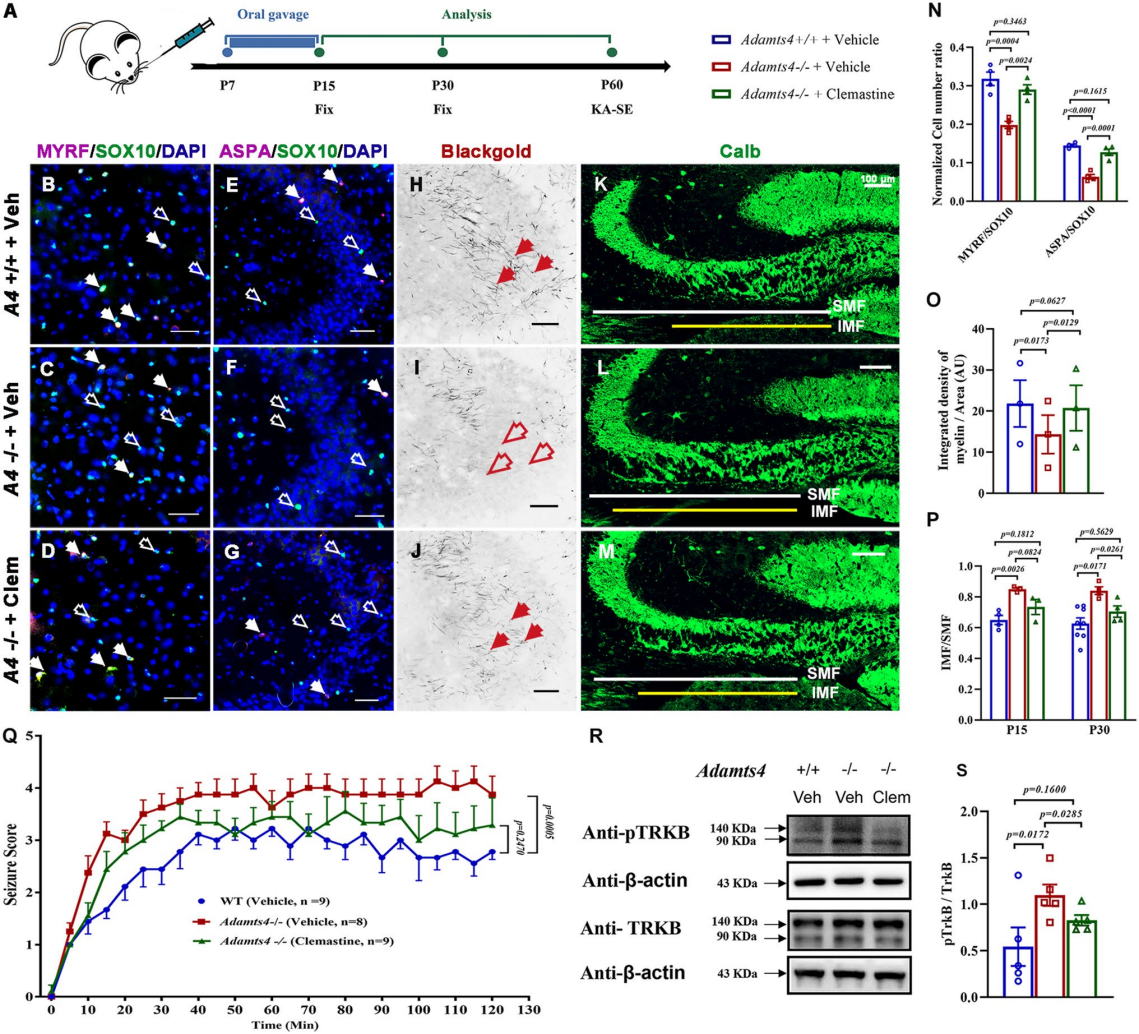
Clemastine improves oligodendrocyte differentiation that influences the epileptic seizure threshold in the *Adamts4* mutant. **A** Experimental scheme. **B–D** Representative images showing co-immunostaining of the pan-oligodendrocyte marker SOX10 (green) with differentiating oligodendrocyte MYRF (magenta) in the CA3 region of *Adamts4*-KO hippocampus treated with vehicle or clemastine at P15. **E–G** Immunofluorescent staining of anti-ASPA and anti-SOX10 in the CA3 region of *Adamts4-*KO hippocampus treated with vehicle or clemastine at P15. Solid arrows indicate SOX10^+^MYRF^+^ or SOX10^+^ASPA^+^ oligodendrocytes; empty arrows indicate SOX10^+^MYRF^–^ or SOX10^+^ASPA^–^ cells. Scale bars: 50 μm. **H–J** Representative images of gold myelin staining in the CA3 region at P15. Scale bars: 50 μm. **K–M** Representative images of immunofluorescence for anti-Calbindin in the hippocampus at P15. Scale bars: 100 μm. **N** The numbers of MYRF^+^ or ASPA^+^ oligodendrocytes per mm^2^ in the hippocampus at P15 and P30. *n* ≥ 3, *P* values are annotated on the bar graphs. **O** The density of myelin fibers per unit of area (μm^2^) in the hippocampus, *n* = 3. *P* values are annotated on the bar graphs. **P** Normalized ratio of IMF to SMF at P15 and P30 in the hippocampus. IMF, infra-pyramidal mossy fiber tract; SMF, supra-pyramidal mossy fiber tract. *n * ≥ 3, *P* values are annotated on the bar graphs. **Q** The behavioral response to kainic acid is improved in mutant mice treated with clemastine compared to vehicle control. Seizure scores are recorded every 5 min after KA-induction (*n = Adamts4*^*+/+*^-vehicle: 9; *Adamts4*^*−/−*^-vehicle: 8; *Adamts4*^*−/−*^-clemastine: 9, *P* values are annotated on the line graph). **R** Immunoblots for the pTRKB and TRKB protein expression in the hippocampus at P15. **S** Statistical analysis of the pTRKB protein expression in mouse hippocampus.

We next investigated whether enhancing OL differentiation is beneficial for abnormal MFs and consequently reduces epileptic susceptibility in mice with hypomyelination. Clemastine is an FDA-approved compound that could cross the blood-brain barrier and acts to promote OLs differentiation both in vitro and in vivo [35, 36]. In this study, clemastine compound (10 mg/kg) was administered to *Adamts4* mutant pups by oral gavage daily from P7 to P15, a critical period for OL differentiation and neural circuitry formation in the hippocampus (Fig. 5A). In the control groups, the wild-type and *Adamts4-*null littermates were treated with ddH_2_O. We discovered that treatment of *Adamts4*-null mice with clemastine indeed led to a significant increase in the number of MYRF^+^/SOX10^+^ differentiating and ASPA^+^/SOX10^+^ mature OLs in the hippocampus compared to the vehicle at P15 (Fig. 5B–G, N). Gold myelin staining also revealed higher myelinogenesis in the hippocampus of clemastine-treated mutant mice (Fig. 5H–J, O).

Meanwhile, we carried out anti-Calbindin immunostaining to assess the MFs in the hippocampus at P15 and P30 (Fig. 5K–M) and found that clemastine treatment significantly reduced the IMF/SMF length ratio of MFs in the mutants compared to the vehicle groups. In the clemastine-treated group, no significant difference in the ratio was found between wild-type and mutant mice (Fig. 5P). For behavior tests, P60 mice were injected intraperitoneally with KA and then recorded every 5 min for 2 h. Based on the modified Racine scale, the mutant mice treated with vehicle ddH_2_O displayed the highest seizure score, whereas the wild-type mice had the lowest score. Importantly, the mutant mice treated with clemastine scores were moderated (Fig. 5Q). These results suggest that clemastine treatment in *Adamts4* mutants significantly rescues the epileptic threshold and consequently reduces the animal susceptibility to epilepsy. Analyses of TRKB phosphorylation by immunoblotting in P15 hippocampus showed that its level was dramatically elevated in the mutant group as compared to the wild-type group, and this elevation was attenuated when clemastine was administered to the mutants (Fig. 5R, S). Together, these findings suggest that clemastine enhances OL differentiation in the developing hippocampus, decreases pTRKB phosphorylation-regulated MF outgrowth, and attenuates the epileptic susceptibility of the mutants.

## Discussion

Oligodendrocytes, the myelinating glia of the central nervous system (CNS), exhibit region-specific functional heterogeneity between white and gray matter, with distinct implications for neurological and psychiatric disorders. In white matter, OLs are primarily responsible for generating multilamellar myelin sheaths to insulate axons, enabling saltatory conduction and maintaining axonal integrity [10]. Their dysfunction disrupts signal transmission efficiency and contributes to demyelinating diseases such as multiple sclerosis (MS) and leukodystrophies, where progressive myelin loss leads to neurodegeneration and motor/cognitive deficits [37]. Notably, recent studies highlight that OL precursor cells in white matter exhibit impaired differentiation in MS, suggesting that myelin repair failure exacerbates disease progression [38]. In contrast, OLs in gray matter are increasingly recognized for their non-myelinating roles, including metabolic support for neurons, modulation of synaptic plasticity, and regulation of ion homeostasis. These cells form synapses with neurons and dynamically influence cortical circuit activity [39].

Gray matter OL dysfunction has been implicated in psychiatric disorders such as schizophrenia and major depressive disorder, where postmortem studies reveal reduced OL density and altered myelin-related gene expression [40, 41]. For instance, disrupted OL-mediated glutamate recycling in gray matter may contribute to the excitotoxicity and synaptic imbalance in schizophrenia. Furthermore, animal models have demonstrated that OL ablation in cortical regions induces anxiety-like behaviors and cognitive impairments, underscoring their role in maintaining emotional and cognitive networks [42]. Emerging evidence suggests that hippocampal OL dysfunction is closely linked to epileptogenesis. In TLE, the most common form of focal epilepsy, pathological hallmarks include hippocampal sclerosis, axonal demyelination, and reactive gliosis [43]. Postmortem studies of TLE patients and animal models have revealed a pronounced loss of OLs and disrupted myelin integrity in the hippocampus, particularly in the dentate gyrus, CA1, and CA3 regions [44]. In this study, we discovered that *Myrf*-CKO (*Olig1*^Cre^; *Myrf* ^fl/fl^) developed seizures and died by P16 (Fig. 1O, P). Knockdown of *Myrf* in OLs resulted in severe deficits in differentiation and failure of myelinogenesis in the hippocampus at P15 (Fig. 1A–O, J, K–N). Also, the deficiency of *Myrf* in OLs led to a decrease of SOX10^+^ pan-OLs in mutant tissue, in association with impeded differentiation. By assessing the hippocampal mossy fiber development, we revealed that a deficiency in hippocampal OL differentiation changed the mossy fiber length ratio (Fig. 1Q–S). Besides, increased GluR1 phosphorylation, which caused abnormal glutamatergic neurotransmission, was found in the *Myrf*-CKO mouse hippocampus (Fig. 1Y, Z).

The hippocampus, a brain region critical for learning, memory, and spatial navigation, harbors a unique population of OLs that contribute to both structural and functional homeostasis. In this region, OLs not only myelinate axons of the perforant pathway and Schaffer collaterals but also engage in bidirectional communication with hippocampal neurons. Hippocampal OL differentiation starts at the early postnatal stage and is most rapid between P14 and P45 in the mouse brain, the critical period of hippocampal neural circuit formation. This suggests that OLs may regulate hippocampal mossy fiber development at the early postnatal stage. To address this possibility, in different mutant mouse lines, we examined *Adamts4*, which is abundantly expressed in differentiating OLs and also regulates OL differentiation [16]. In contrast to *Myrf*-CKO mice that succumb to epilepsy by P15, *Adamts4*-KO mice remain viable. Here, we showed the association between deficient hippocampal OL differentiation and abnormal mossy fiber development in *Adamts4-*null mice (Fig. 3A–C, H–J). The normalized ratio of IMF to SMF is commonly used for assessing MF development during hippocampal microcircuit formation [8]. Consistent with the aberrant mossy fibers in the hippocampus, the *Adamts4*-null mice exhibited higher epileptic behavioral scores than the controls, becoming more susceptible to epilepsy (Fig. 3K). Our previous work demonstrated that *Secisbp2l*, specifically expressed by OLs, influences the differentiation of OLs [45]. Consistent with this, defective OL differentiation in *Secisbp2l*-cKO mice altered the hippocampal mossy fiber length ratio and lowered the threshold of epileptogenesis (Fig. S3). Thus, retarded OL differentiation impairs mossy fiber development in the hippocampus at early stages and increases seizure susceptibility in adults even though myelin is gradually restored at later stages (Fig. 2D–G), in keeping with the phenotypes of *Adamts4*-KO mice.

**Fig. 6:**
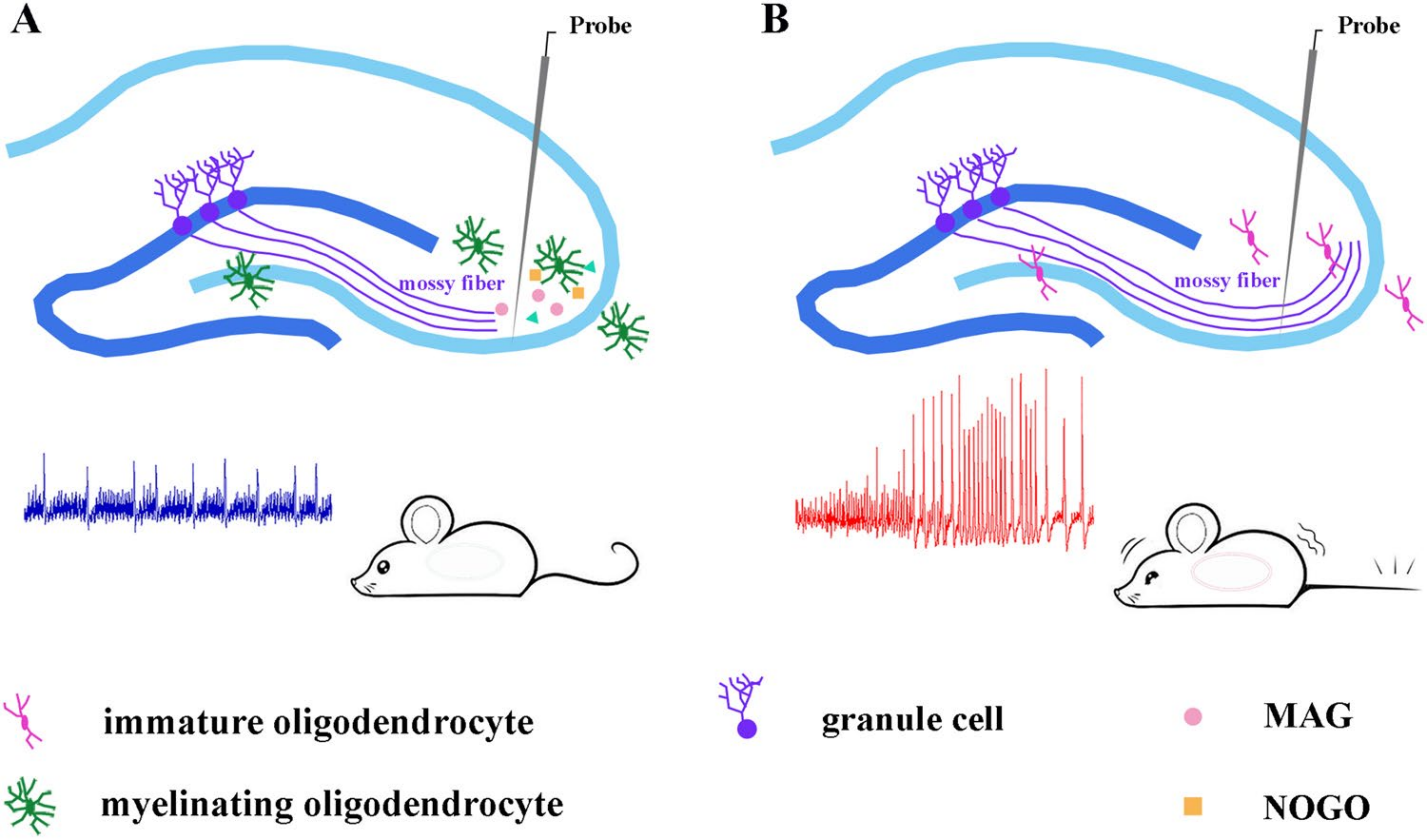
Proposed molecular model of how oligodendroglial development affects hippocampal mossy fiber specification with an impact on epilepsy. In the hippocampus, mature OLs extend processes to wrap the axon and engage in bidirectional communication with hippocampal neurons. Meanwhile, some inhibitory factors are produced by OLs (i.e. MAG, NOGO), which inhibit neurite outgrowth. Correspondingly, a significant down-regulation of MAG/NOGO expression in the hippocampus of mice with defective OL differentiation attenuates their inhibitory effect on axon growth. These mice exhibit abnormal mossy fiber development (i.e. abnormal hippocampal neural circuitry), leading to a higher susceptibility to epileptic seizures.

It has been demonstrated that the inhibitors NOGO and MAG inhibit axon growth in the CNS [29, 30]. MAG, a protein whose expression is upregulated during OL differentiation, is predictably reduced in the *Adamts4* and *Myrf*-CKO mice (Fig. 4A–H). More importantly, MAG inhibits neurite outgrowth by binding paired immunoglobulin-like receptor B with neurotrophin receptor tyrosine kinase (TRKB), which further recruits SHP1 and SHP2 to dephosphorylate pTRKB [34]. In other words, the loss of MAG leads to increased expression of phosphorylated TRKB and relieves the inhibition of axon outgrowth. During development, axons of granule cells in the dentate gyrus project to pyramidal cells in the CA3 region to form synapses. The mossy fibers are the axons of granule cells, which are divided into infrapyramidal mossy fibers (IMFs) and suprapyramidal mossy fibers (SMFs). Generally, newly-formed axons are preferentially added to the IMFs. The increased length ratio of mossy fibers in the hippocampus of mice means longer axons and abnormal neural circuits [8, 46]. Consequently, mice with retarded OL differentiation displayed enhanced excitatory electrophysiological features (Fig. 2N, O). In addition, we found a significant up-regulation of pTRKB in mice with abnormal OL development caused by diminished dephosphorylation (Fig. 4I, J). It has been demonstrated that hyperactivation of TRKB and its downstream signaling molecules increases presynaptic vesicle release and enhances excitatory synaptic transmission, leading to epileptogenesis [47]. Therefore, we proposed that down-regulated MAG, consistent with abnormal hippocampal OL differentiation, affects pTRKB dephosphation and subsequently attenuates the inhibition of axon outgrowth that eventually causes susceptibility to epilepsy in mice (Fig. 6). Inspiringly, drug-enhanced OL differentiation in *Adamts4* mutants at early postnatal stages restores the hippocampal neural circuit, leading to an increased threshold for epilepsy compared to vehicle-treated mutants. These findings provide additional supporting evidence that OLs play a significant role in hippocampal mossy fiber construction and epileptogenesis. However, the current models are unable to effectively achieve region-specific enhancement of OL differentiation in the hippocampus to rescue the epileptic phenotype. It is also hard to evaluate the contribution to the occurrence of epilepsy that comes from brain regions outside of the hippocampus. To address these methodological constraints, it is imperative to develop hippocampal-specific models that enable precise spatiotemporal modulation of OL differentiation dynamics and subsequent myelination.

In summary, we discovered that decreased mature OLs in the young hippocampus can cause abnormal mossy fiber growth and a higher susceptibility to epilepsy in adult mutant mice. Remarkably, the enhancement of OL differentiation with the drug clemastine alleviated these phenotypic alterations in the mutants, providing a novel therapeutic target and strategy for preventing epileptogenesis. In addition, this study demonstrates that hippocampal OLs play an important role in the construction of hippocampal neural circuits during the early postnatal period and the epileptic responses at the adult stage.

## Supplementary Information

Below is the link to the electronic supplementary material.

Supplementary file1 (PDF 704 kb)

Supplementary file2 (MP4 5834 kb)

## Data Availability

The data that support the findings of this study are available from the corresponding author upon reasonable request.
