# Supplementary material for: Hippocampal Oligodendrocytes Regulate Mossy Fiber Development Involved in Epileptic Responses
Source: Neurosci Bull. 2025 Jul 17;42(4):781–94. doi: 10.1007/s12264-025-01452-x (PMC13031610; doi:10.1007/s12264-025-01452-x)
Supplement: Supplementary file 1 — Supplementary file1 (PDF 704 kb) [file 12264_2025_1452_MOESM1_ESM.pdf]

Supplementary information:

Supplementary Video

Video for continuous real-time recorded *Olig1*^{Cre}; *Myrf*-CKO mice behavior.

Figure S1-S3

Figure_1_SuppInfo.

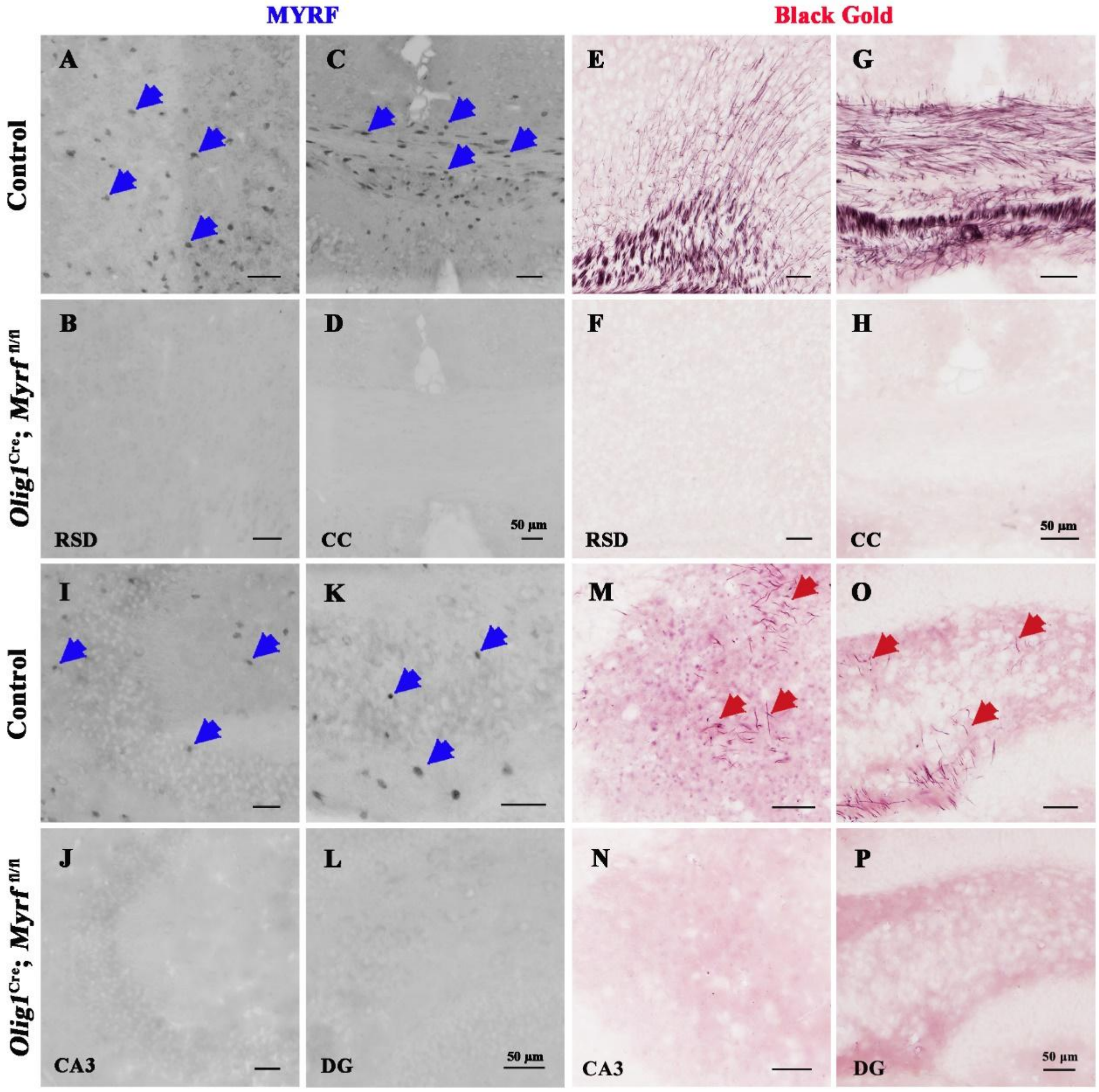

***Myrf* ablation affected oligodendrocyte differentiation and myelin formation. A-D.** Immunofluorescent staining of anti-MYRF in the RSD (retrosplenial dysgranular cortex) (**A-B**) and CC（Corpus callosum）(**C-D**) of *Olig1* ^{Cre}; *Myrf* CKO mice at P14. Scale bar: 50 µm. **E-H.**

Representative pictures for gold myelin staining in the the RSD (retrosplenial dysgranular cortex) (**E-F**) and CC（Corpus callosum）(**G-H**) of *Olig1* ^{Cre}; *Myrf* CKO mice at P14. Scale bar: 50 μm. **I-L.** Immunofluorescent staining of anti-MYRF in the CA3 (**I-J**) and DG (**K-L**) of *Olig1* ^{Cre}; *Myrf* CKO mice hippocampus at P14. Scale bar: 50 μm. **M-P.** Representative pictures for gold myelin staining in the CA3 (**M-N**) and DG (**O-P**) of *Olig1* ^{Cre};*Myrf* CKO mice hippocampus at P14. Scale bar: 50 μm.

Figure_2_SuppInfo.

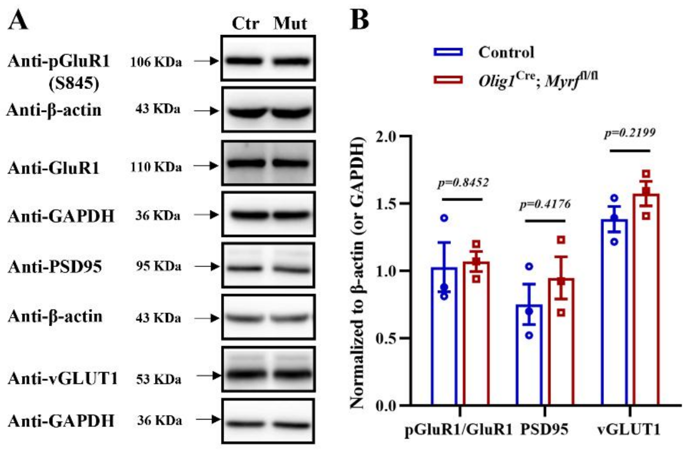

Myrf **deficiency did not influence the level of pGluR1 in cortex. A-B.** Western immunoblot for Glutamate receptor 1 (GluR1) serine phosphorylation and total protein expression levels of GluR1, PSD95 and vGLUT1 (**A**) quantified in (**B**). n = 3, p values were annotated on the bar graphs.

Figure_3_SuppInfo.

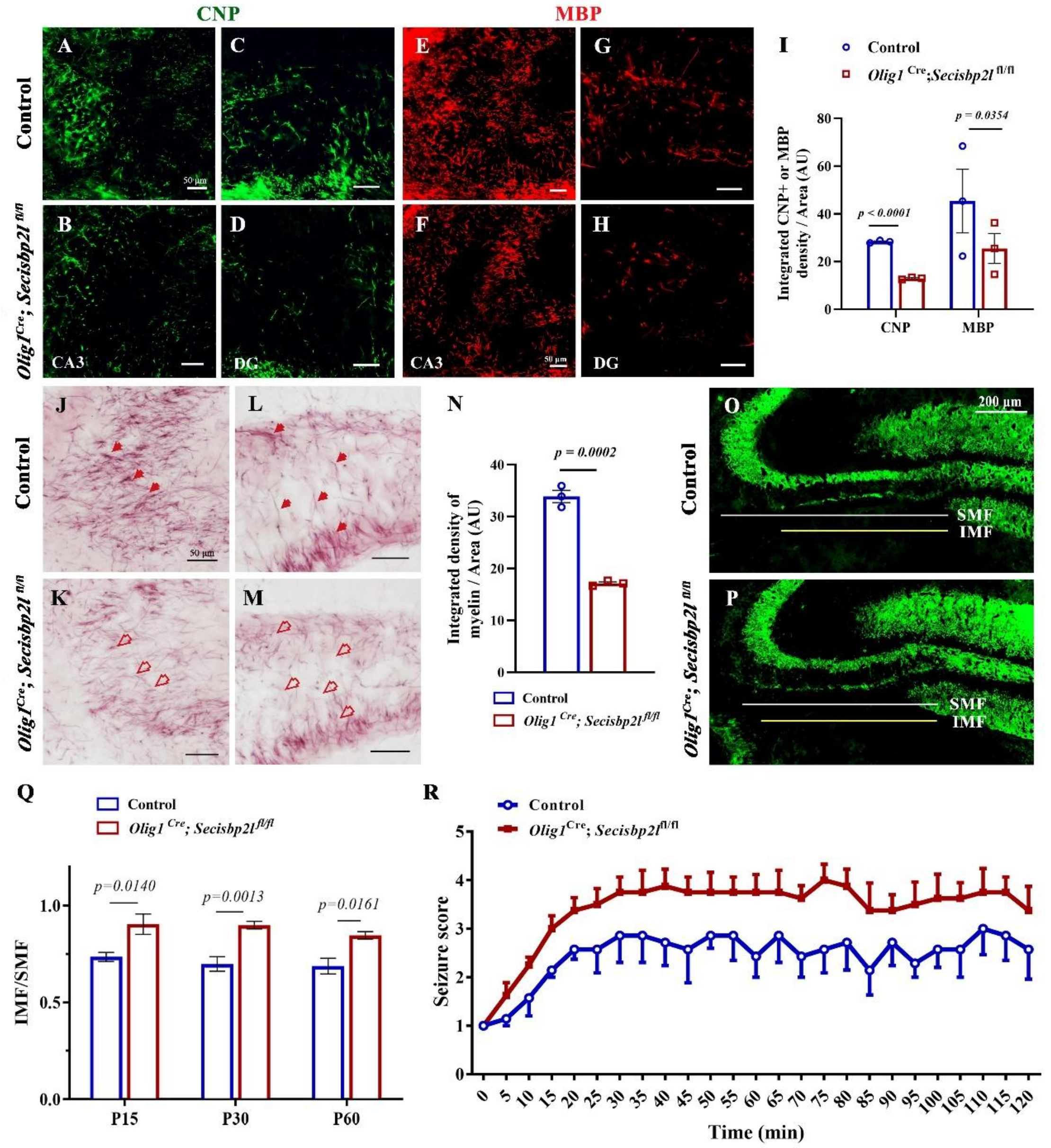

***Secisbp2l* deletion influence oligodendrocyte differentiation, mossy fiber length ratio and mice behavioral response to epilepsy.** A-H. Immunofluorescence staining against anti-CNP(A-D) and anti-MBP(E-H) in *Olig1* ^{Cre}; *Secisbp2l*-cKO mice hippocampus at P15. Scale bar: 50 μm. I. Statistical analysis of the density of CNP or MBP immunostaining per unit of area (μm ^{2}) at P15. AU, Arbitrary Units. n = 3. *p* values were annotated on the bar graphs. J-M. Gold myelin staining kit for axonal myelin sheath in the hippocampus of *Olig1* ^{Cre}; *Secisbp2l*-cKO mice at P30. Scale bar, 50 μm. N. Statistical analysis of the density of myelin fibers per unit of area (μm ^{2}), n = 3. *p* values were annotated on the bar graphs. O-P. Immunofluorescence of anti-Calbindin in the hippocampus of *Olig1* ^{Cre}; *Secisbp2l*-cKO mice at P15. IMF, infra-pyramidal mossy fiber tract; SMF, super-pyramidal mossy fiber tract. Scale bar: 200 μm. Q. Normalized ratio of IMF to SMF at P15 to P60 in *Olig1* ^{Cre}; *Secisbp2l*-cKO mice hippocampus (n = 3, p values are shown in each group). R. Behavioral response to KA-induced seizure was altered in *Secisbp2l* mutant mice. Seizure scores

were recorded every 5 min for control and *Olig1* ^{Cre}; *Secisbp2l* $^{fl/fl}$ mice after KA induced (n=Control: 8; Mutant: 10).
